# Vitamin D in Early Childhood and the Effect on Immunity to *Mycobacterium tuberculosis*

**DOI:** 10.1155/2012/430972

**Published:** 2012-07-05

**Authors:** Anna Jane Battersby, Beate Kampmann, Sarah Burl

**Affiliations:** ^1^Academic Department of Paediatrics, Imperial College London, St. Mary's Campus, Wright Fleming Building, Norfolk Place, London W2 1PG, UK; ^2^Infant Immunology, Medical Research Council Unit, The Gambia, Atlantic Boulevard, Fajara, Gambia

## Abstract

A potential role for vitamin D as a therapeutic immunomodulator in tuberculosis (TB) has been recognised for over 150 years, but has only recently returned to the centre of the research arena due to the increasing awareness of the global vitamin D deficiency epidemic. As early as birth a child is often deficient in vitamin D, which may not only affect their bone metabolism but also modulate their immune function, contributing to the increased susceptibility to many infections seen early in life. Recent studies have begun to explain the mechanisms by which vitamin D affects immunity. Antimicrobial peptides are induced in conjunction with stimulation of innate pattern recognition receptors enhancing immunity to particular infections. In contrast the role of vitamin D within the adaptive immune response appears to be more regulatory in function, perhaps as a mechanism to reduce unwanted inflammation. In this paper we focus on the effect of vitamin D on immunity to TB. Where much of the attention has been paid by past reviews to the role of vitamin D in adult TB patients, this paper, where possible, focuses on research in paediatric populations.

## 1. Introduction

Immune responses to *Mycobacterium tuberculosis (MTB)* are complex and remain incompletely understood. However, with recent advances in the field of immunology, we have learnt more about how *MTB* infects the human host and, in turn causes disease. There is increasing epidemiological evidence to support the role of vitamin D in the immune response to tuberculosis (TB) [1]. A recent meta-analysis included 7 studies with 531 participants and reported that low serum vitamin D levels were associated with a higher risk of active TB [1]. Additionally, an association of TB with season has been observed in many countries, including the UK where the incidence of TB is greater in the spring/summer months. The decreased vitamin D levels in the spring are thought to follow reduced sun exposure during winter months (the circulating form of vitamin D, 25-hydroxyvitamin D has an average half-life of 2–8 weeks [2–5]). Similar seasonality of TB [6] has been noted in Europe [4, 7], South Africa [8, 9], and India [10]. Dietary factors also appear to influence vitamin D status and susceptibility to TB. In a study of Asian UK immigrants, the vegetarian diet, which is known to be low in vitamin D, was an independent risk factor for TB [11]. The mechanisms by which vitamin D may help to prevent or clear MTB infection and/or active TB are not completely clarified to date, but studies that have helped in the understanding of its role will be discussed in this paper.

## 2. Historical Context: The Road to Rediscovery

Cod-liver oil was traditionally used in the treatment of tuberculosis in the late nineteenth and early twentieth centuries [12]. The earliest case reports describing the effects of cod-liver oil in TB appeared in 1846 [13] and were followed subsequently by numerous cases that supported the notion that this dietary supplement could provide demonstrable improvements in the health of TB sufferers [14]. Later in the nineteenth century, patients were frequently treated in sanatoriums, which were built in the countryside, and were designed to provide sufferers with therapeutic “fresh air” and notably, sunshine. Indeed the clinical use of sunlight exposure or “heliotherapy” gained significant momentum following the award of the Nobel prize for medicine in 1903 to Niels Ryberg Finsen: “*in recognition of his contribution to the treatment of diseases, especially lupus vulgaris (tuberculosis of the skin), with concentrated light radiation, whereby he has opened a new avenue for medical science”* [15]. 

It was not until later that vitamin D was discovered as the active ingredient in cod-liver oil [17]. Charpy, a physician from Dijon, France, appears to be one of the earliest individuals to effectively implement the clinical use of vitamin D_2_ (calciferol) [18]. In 1945, he reported successfully using the formulation to treat 20 patients with lupus vulgaris. He obtained some “remarkable results” which were then reproduced by others in Europe and around the world [18–20]. By 1946 in London, Dr. Dowling and his colleagues had also used calciferol on a number of patients. They reported in the *Proceedings of the Royal Society of Medicine *that their experience could *“leave no room for doubt that calciferol in adequate dosage will cure a substantial proportion of cases of lupus”* [20].

The first reference to successful treatment of pulmonary TB with vitamin D appeared in the Lancet in 1947 [21]. The discovery by Alexander Fleming in 1928 of penicillin and its subsequent mass production and distribution by 1945 revolutionised medical treatment of infectious diseases, although not specifically TB, yet it appears that the benefits of vitamin D were somewhat overlooked in the wake of the antibiotic era [22].

## 3. Vitamin D Biochemistry

The term vitamin D encompasses a number of steroid-like proteins: vitamins D_2_–D_7_. Vitamins D_2_ and D_3_ have known physiological significance in humans, with both undergoing hydroxylation steps to become active hormones in calcium and phosphate metabolism [23]. Their chemical structure is based on 4 steroid rings; 1 of which is broken. Vitamins D_2_ and D_3_ differ only by the nature of their side chains [24]. The 2 forms of vitamin D can be obtained from the diet, but predominantly, vitamin D is obtained in the D_3_ form, from the action of UV light on a vitamin D precursor in the skin [25].

Vitamin D_3_ undergoes two hydroxylation steps before becoming an active hormone: the first step occurs in the liver and results in the production of 25-hydroxyvitamin D (25[OH]D) [26]. The “25” of 25-hydroxyvitamin D refers to the location of a hydroxyl group on one of the side arms of the steroid rings. This form of vitamin D must undergo a further hydroxylation step to become physiologically active in the form of 1-*α*,25-hydroxyvitamin D (1*α*,25[OH]2D). In the past, it had erroneously been assumed that this second hydroxylation step was only performed by the kidneys; however, it is now clear that a number of cells, particularly innate immune cells such as monocytes and macrophages, possess the machinery required to produce active 1*α*,25[OH]2D. Indeed, since the discovery of vitamin D receptors (VDRs) in macrophages, the role of 1*α*,25[OH]2D as an immune modulator has become increasingly apparent [27].

In children, and neonates in particular, a C3-epimer of the 25[OH]D molecule, 3-epi-25[OH]D_3_, often constitutes a significant proportion of the total circulating 25[OH]D [28, 29]. It is therefore important, although not universal practice, to identify the proportion of the 3-epi-25[OH]D_3_ when measuring 25[OH]D levels in children. The only method to reliably do this is the liquid chromatography-tandem mass spectroscopy method (LC-MS/MS) [28] enabling the epimer measurement to be removed from the final result if required. The RIA (radioimmune assay) method does not react with the epimer and the high performance liquid chromatography (HPLC) method cross-reacts with the epimer and causes interference without being able to discriminate between the isoforms making these methods unreliable when measuring vitamin D levels in infants [28]. The 3-epi-25[OH]D_3_ differs from 25[OH]D by the asymmetrical arrangement of a hydroxyl group at the C3 position [28]. It is thought that this epimer may be the result of immature vitamin D metabolism and may display reduced efficacy in calcium-mediated bone metabolism [30]. Interestingly, the 3-epi-25[OH]D_3_ form of vitamin D has a lower binding affinity for the VDR. However, this does not necessarily translate into reduced biological effects [31–33]. The overall impact of the 3-epi-25[OH]D_3_ on immune health in infancy remains unknown. However, we can speculate that supplementation may be less effective in the infant cohort, because when the vitamin D supplement (cholecalciferol or ergocalciferol) undergoes the first hydroxylation step, the infant produces a large proportion of a physiologically less effective epimer of vitamin D, 3-epi-25[OH]D_3_. This may help to explain why a recent large-scale vitamin D supplementation trial in preterm neonates reported no significant effect on overall morbidity and mortality [34].

## 4. What Constitutes Vitamin D Sufficiency?

There is no agreed consensus on the optimal level for vitamin D status in the adult [26, 35–38] and particularly what constitutes vitamin D sufficiency in childhood. In the UK there are no up-to-date guidelines to define deficiency and insufficiency [36]. It is true that with 25[OH]D levels below <25 nmol/L children manifest clinical signs and symptoms of rickets [37]. However, children can be diagnosed with clinical rickets at higher 25[OH]D levels. Levels of 25[OH]D above the 25 nmol/L cut-off may be associated with other poor health outcomes [39], such as upper respiratory tract infections [40] and bronchiolitis [41]. A recent paper in the British Medical Journal summarises current opinion regarding adult vitamin D endocrine levels (Table 1) [37]. However, as mentioned previously the situation is further complicated in infancy by the presence of the C3-epimer of 25[OH]D which can contribute to erroneously high total 25[OH]D levels [28].

With increasing knowledge of the endocrine functions of vitamin D and more recent evidence of possible autocrine/paracrine functions, it is now important to also consider the concentration of vitamin D required to drive an appropriate immune response. At present this is not known but *in vitro* experiments suggest that at 98 nmol/L concentration IFN*γ* can induce antimicrobial expression and can reduce growth of *Mycobacterium tuberculosis *whereas levels of 45 nmol/L cannot [42].

Physiological ranges for circulating 25[OH]D_3_ can extend to beyond 200 nmol/L which is much greater than the considered average “norm” for a population. As a perspective, concentrations of 25[OH]D in nonhuman primates have a median value of 170 nmol/L and a lowest value >80 nmol/L [38], whereas modern humans in winter have a median value of 40 nmol/L and a maximum value of 70 nmol/L [38], a similar amount to that of rodents. In addition, a recent study of Masasai and Hadzabe hunter-gatherer traditional populations in Tanzania showed that the mean serum levels of 25[OH]D were 119 and 109 nmol/L, respectively, and none were below 50 nmol/L. These higher levels, only seen in Caucasian lifeguard populations that were exposed to more than 3 hours of sun per day for more than 5 day/week for at least 3 months, may serve as targets for further research [43].

With this lack of agreement on what levels of 25[OH]D constitute sufficiency, in turn there is variability in recommendations for supplementation. However a US study of newborns found that 78% had levels of 25[OH]D < 75 nmol/L and 17% had levels < 30 nmol/L suggesting a need for supplementation from birth [44]. The US Endocrine Society proposes that infants and children aged 0-1 year require at least 400 IU per day of vitamin D and that children 1 year and older require at least 600 IU per day to maximize bone health [5]. A recent study showed that in pregnant women supplementation of 4000 IU/d was safe and most effective in achieving sufficiency in women and their neonates, whereas the current estimated average requirement (200–400 IU/d) is comparatively ineffective at achieving adequate circulating 25[OH]D concentrations [45]. In the UK vitamin D supplementation for all mothers of breastfed infants is recommended and in infants greater than 6 months who are taking less than 500 mL of formula milk per day [46]. US guidelines recommend that supplementation directly to the breast fed (or partially breast fed) infant should commence in the first few days of life [25]. Partly because of the lack of agreement between health professionals, but also for a plethora of other reasons, compliance to these supplementation recommendations across countries remains poor.

## 5. Vitamin D as an Immunomodulator

The role of 1*α*,25[OH]2D in calcium and phosphate metabolism and bone health has been long established, but its immunomodulatory function remains poorly defined. In addition to TB as mentioned earlier, there is growing evidence that lower 25[OH]D levels are associated with a higher incidence of other infections, particularly of the respiratory tract [40, 52–55]. However, there remains a large degree of uncertainty in this area; for example, some studies find that vitamin D deficiency is associated with worse severity of infection in childhood [53, 56, 57], whilst others do not [58]. To help understand these discrepancies, the role of vitamin D in immunity, particularly with regards to TB, is further discussed.

**Figure 1:**
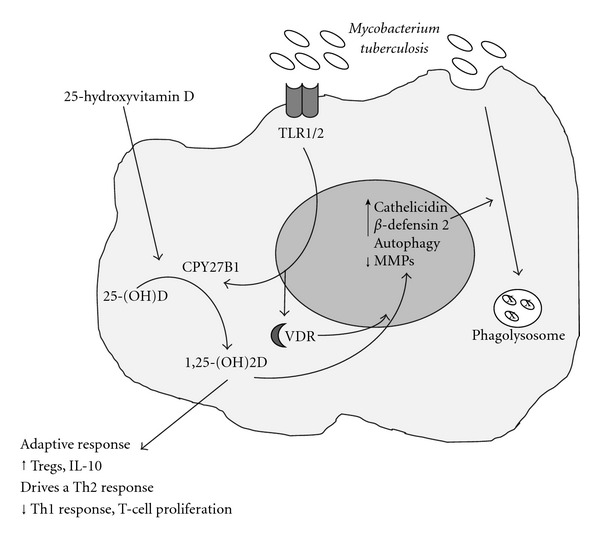
Mechanism of vitamin-D-induced immunity to *Mycobacterium tuberculosis* (modified from [16]). Stimulation of monocyte Toll-like receptors (TLR1/2) by *Mycobacterium tuberculosis* (MTB) results in transcriptional induction of the vitamin D receptor (VDR) and 1*α*-hydroxylase (CYP27B1). Circulating 25-hydroxyvitamin D (25[OH]D) enters the cell and is converted to 1,25-dihydroxyvitamin D (1,25[OH]2D) by the CYP27B1 enzyme. VDR-bound 1,25(OH)2D then induces expression of cathelicidin and *β*-defensin 2 (DEFB4). In addition 1,25(OH)2D induces autophagy and downregulating metalloproteinases (MMPs), all of which help in the formation of phagolysosomes and the killing of Mtb. 1,25(OH)2D also affects the adaptive immune system and leads to an upregulation of regulatory responses and a skewing towards a Th2 response. IFN*γ* is thought to induce the expression of the CYP27B1 enzyme suggesting a feedback mechanism between the innate and adaptive response to vitamin D.

There is significant biological plausibility for a clinical association between low 25[OH]D levels and infection with many studies describing the direct effect of 1*α*,25[OH]2D on innate immunity [16, 59, 60]. Many immune cells express the VDR, including T and B cells [61], dendritic cells [62], as well as macrophages [63]. Initial studies found that 1*α*,25[OH]2D stimulates antimicrobial activity [57, 58], but it is only recently that the possible mechanism has been described. Ligation of the innate immune pattern recognition receptors, Toll-like receptors (TLRs) on human macrophages, causes upregulation of the intracellular VDR and vitamin D_1_ hydroxylase genes, resulting in induction of cathelicidin [64] and/or *β* defensin [65], both of which are potent antimicrobial peptides (Figure 1). It appears that this action of 1*α*,25[OH]2D may be dependent on the presence of interferon gamma (IFN*γ*) [42, 66] suggesting a link with adaptive immunity.

In 1986 Rook et al. found that incubation of monocytes with 1*α*,25[OH]2D inhibited growth of *MTB *[67]. Although this appeared to be independent of IFN*γ*, the addition of IFN*γ* along with 1*α*,25[OH]2D resulted in a synergistic effect on mycobacterial growth inhibition [67]. In addition Denis showed that in the presence of IFN*γ* and TNF*α*, 1*α*,25[OH]2D promoted increased intramonocyte killing of *MTB *[68]. These effects are thought to be exerted through the release of antimicrobial peptides as described previously [65, 69]. Indeed, the more recent paper by Liu et al. in 2006 shows that monocyte TLR ligation promotes conversion of 25[OH]D to 1*α*,25[OH]2D and subsequent cathelicidin release. Cathelicidin induces fusion of the phagolysosome, which is essential for the containment, degradation and subsequent killing of *MTB *[64] (Figure 1).

It seems that 1*α*,25[OH]2D also exerts its effects on innate immune responses by the promotion of autophagy [70–72] and the suppression of tissue remodelling and lung matrix breakdown [73]. Autophagy is a potent mechanism by which the host defends against mycobacterial infection, by degradation of a cells' own components through the lysosomal machinery [74]. Fabri and Modlin have shown that monocytes cultured in vitamin D sufficient sera and stimulated with IFN*γ* display autophagy as well as secrete antimicrobial properties against *MTB *[72]. It has been shown *in vitro* that 1*α*,25[OH]2D downregulates matrix metalloproteinases (MMPs) and upregulates tissue inhibitor of metalloproteinase 1 (TIMP1) in peripheral blood mononuclear cells (PMBCs) in the presence of live *MTB *[73]. *MTB* induces significant pathological effects through tissue remodelling and breakdown of extracellular matrix in the lung, and therefore it is possible that 1*α*,25[OH]2D may protect the host against this effects.

It appears that downstream adaptive immunity can also be modified by 1*α*,25[OH]2D [27, 75, 76], which is evidenced by its effects on human B-cell differentiation [77] and antigen presentation [78]. Interestingly 1*α*,25[OH]2D has antiproliferative effects on CD4+ T cells [79] and appears to inhibit Th1 cytokine production [80–83], whilst promoting T regulatory function [84], and potentially upregulating Th2 cytokine production [85]. Culturing peripheral blood mononuclear cells from TB patients in the presence of 1*α*,25[OH]2D, Vidyarani et al. recently showed that 1*α*,25[OH]2D suppressed IL-12p40 and IFN*γ* production in response to *MTB *antigens [83]. However, for *MTB* to be maintained in a latent state, Th1-cytokine-driven granuloma formation is actually required, and inhibition of Th1 cytokines could therefore be detrimental to the host. Indeed, a recent study using *in vitro* analysis of sera of patients with pulmonary TB has shown that coculture of T cells with 1*α*,25[OH]2D reduces the number of Th1 cytokine expressing cells (specifically IFN*γ* and TNF*α*) [86]. The authors propose that 1*α*,25[OH]2D “may play a dual role in the immunity against tuberculosis by eliminating infection as well as reducing inflammation at the site of infection”.

The effects of vitamin D on Th2 cytokine responses are less well understood, and certainly how changes in the Th2 cytokine profile may affect *MTB *infection in humans has not been extensively studied. We do know that the classical Th2 cytokines, IL-4, and IL-13 are potent inhibitors of autophagy, which is an essential immune pathway in the host defence against *MTB* infection although in contrast autophagy has been shown to be induced by vitamin D through the innate response as mentioned earlier [64–66]. Animal studies suggest that production of IL-4, is upregulated in the presence of 1*α*,25[OH]2D [85]. Indeed, Boonstra et al. [87] have demonstrated that 1*α*,25[OH]2D induces Th2 cell development and IL-4, IL-5, and IL-10 production *in vitro*. However, it is prudent to say that the effects of vitamin D on Th2 cytokine production remain unclear [88], and how changes to the Th2 cytokine profile may affect the pathophysiology of *MTB* infection needs further elucidation.

## 6. Vitamin D and Infant Immunity to TB

In children, infections remain a major cause of morbidity and mortality around the world [89, 90]. Many epidemiological studies have shown that vitamin D is associated with respiratory disease and viral infections including HIV [91]. There is very little literature describing the role of vitamin D in immunity to TB in infants one such paper examined vitamin D status in children with active TB and found that 86% were vitamin D deficient (25[OH]D < 20 nmol/L) or insufficient (25[OH]D < 75 nmol/L) [92].

The predominant cytokine essential for mycobacterial immunity is IFN*γ* as shown in studies of patients who lack the IFN*γ* and IL-12 receptor that leads to a predisposition to mycobacterial infections [93]. The dual role of IFN*γ* in enhancing the effects of vitamin D *in vitro,* although, being in itself reduced by addition of vitamin D, may be quite different when considering a neonate. In neonates NK production of IFN*γ* has a dominant role to play in response to* MTB* antigens rather than T cells as observed in older infants and adults [94]. There are many other distinct qualities of the immature immune response, in particular reduced Th1 adaptive responses and attenuated innate immunity [95, 96] which suggests that vitamin D may have different effects in the immune system of infants than that of adults. *In vitro* supplementation of 25[OH]D in cord blood cultures showed increased TLR-induced cathelicidin expression suggesting that supplementation in neonates may improve antimicrobial activity [44]. It is known that infants and young children are particularly susceptible to severe TB [97, 98] and therefore the role of vitamin D in childhood TB warrants further investigation.

Trying to understand the varying effects of BCG worldwide one study has looked at the association of vitamin D with BCG vaccination. Lalor's recent observational study of UK infants found that those that were BCG vaccinated had higher vitamin D levels at 3 and 9 months of age, compared to unvaccinated controls [99]. Interestingly infants with higher vitamin D levels had lower IFN*γ* responses to the *Mycobacterium-tuberculosis-*purified protein derivative (*MTB *PPD). As previously discussed*, in vitro*, 1*α*,25[OH]2D) has been shown to have dual effects by, on the one hand, dampening Th1 responses [81], yet on the other stimulating antibacterial peptide secretion to aid clearance of *MTB *[64]. Traditionally IFN*γ* response to vaccination is used as an indicatory of the effectiveness of the vaccine. However, Lalor suggests that in the context of BCG vaccination, a dampened IFN*γ* response may be beneficial to the host, in preventing unnecessary inflammation in response to the *Mtb* PPD which needs to remain present to provide protection against TB [99]. A recent animal study supports this finding with reduced IFN*γ* and IL-17F gene expression in PPD-stimulated blood of BCG vaccinated cattle after addition of vitamin D [100].

## 7. It Is in the Genes

Genetic factors clearly play a role: specific vitamin D receptor (VDR) polymorphisms are associated with a higher risk of TB [101]. The association between TB incidence and other polymorphisms varies widely across different ethnic groups. For example, the *FokI ff* genotype of the VDR appears to be most consistently associated with increased susceptibility to TB among Asians, but not Africans [102], whereas the *tt* genotype of *TaqI* has been shown in Gambian men to be associated with a higher risk of TB [103]. No studies have looked at VDR polymorphisms and incidence of TB in children, but a recent Canadian study reported an association between the *FokI ff* genotype of the VDR and acute lower respiratory tract infection in young children [104]. 

## 8. Vitamin D Supplementation and TB Treatment

There are disparate reports in the literature regarding a role of vitamin D supplementation in the treatment of TB infection (Table 2). Early studies reported a favourable response [47, 51] to vitamin D supplementation. In Jakarta in 2006, Nursyam et al. randomised 67 patients and found that 100% of the vitamin D group versus only 76.7% of the placebo group had sputum conversion at 12 weeks (*P* = 0.002) [47]. However, Wejse et al. found no effect of supplementation on disease outcome [49]. The study addressed the use of 100,000 IU of vitamin D given as an adjunctive treatment at the time of commencement of anti-TB therapy, and again at 5 and 8 months after starting treatment. The authors note that the dose may have been insufficient, and the response to vitamin D dependent on the immune status of the individual patient [49].

A recent large-scale trial of vitamin D supplementation in the treatment of adult TB was carried out by Martineau and colleagues in London, UK [50]. Patients who were receiving standard anti-TB chemotherapy and had been supplemented with vitamin D displayed sputum clearance almost 1 week earlier than those taking placebo (from 43.5 to 36.0 days). However the difference between the intervention and control group did not reach statistical significance. Intriguingly, in a subset of patients with the *TaqI* VDR polymorphism, time to sputum conversion was significantly quicker than that in controls, indicating that these patients would benefit from supplementation.

Prevention of TB with vitamin D supplementation is still debated [13]. In one study TB contacts were given a single dose of 100,000 IU vitamin D. *In vitro* analysis revealed those receiving the supplement had enhanced immunity to mycobacteria, demonstrated through ability of participants' whole blood to restrict luminescence in the BCG-lux assay [95]. These promising laboratory results need to be addressed in a Randomised Control Trial (RCT) to fully support these results. However, there is evidence that vitamin D supplementation can have beneficial effects on immune health in general, and particularly in childhood. a RCT in school children 6–18 years of age showed that oral vitamin D supplementation reduced the incidence of influenza A [92] and a further trial found promise in the use of vitamin D supplementation in the prevention of recurrent pneumonia in children between the ages of 1–36 months [105]. However no specific recommendations exist for the use of vitamin D supplementation to improve immune health outcomes in childhood.

## 9. Future Directions

Recent reports suggest that vitamin D deficiency has become a global health epidemic with 20–100% of US, Canadian, and European elderly men and women being vitamin D deficient [106]. Children and younger adults are equally at risk with vitamin D deficiency common in Australia, the Middle East, India, Africa, and South America [106]. Historically humans probably obtained their vitamin D requirement from prolonged sunlight exposure as “hunters and gatherers”. However, current lifestyle practices, particularly in the developed world, often dictate infrequent sun exposure and a subsequent propensity to deficiency, which is confounded by diets low in vitamin D.

With the expanding evidence that vitamin D not only affects bone metabolism but also may play a large role in immune modulation, these global statistics are worrying. Even neonates at birth are often deficient in vitamin D and if vitamin D can affect immunity to many infectious diseases including TB, then further research in this area is required.

A number of RCTs are currently under way to assess the role of vitamin D in the treatment of tuberculosis and the results are eagerly awaited [107–113]. Of particular interest for child health professionals is the ongoing study in California to determine whether a single oral dose of vitamin D given to infants prior to BCG vaccination will enhance the immune response to BCG vaccination [114].

This paper draws attention to the lack of quality research exploring the role of vitamin D in TB or other infections in early childhood. As we now begin to understand the differences in immune function between adults and children more must be done to address this cohort with respect to vitamin D deficiency and potential benefits of vitamin D supplementation.

## Figures and Tables

**Table 1:** Serum 25-hydroxyvitamin D (25[OH]D) concentrations, health, and disease (modified from [37]).

25[OH]D concentration	Vitamin D status	Manifestation	Management
<25 nmol/L	Deficient	Rickets, Osteomalacia	Treat with high-dose calciferol
25–50 nmol/L	Insufficient	Associated with disease risk	Vitamin D supplementation
50–75 nmol/L	Adequate	Healthy	Lifestyle advice
>75 nmol/L	Optimal	Healthy	None

**Table 2:** Vitamin D single dose (dosage concentration not reported).

Subjects (*n*)	Country	Vitamin D supplement	Findings	Reference
*Adult studies*				
TB patients with pulmonary TB aged 15–59 (67)	Jakarta, Indonesia	0.25 mg per day for 6 weeks	100% of vitamin D group had sputum conversion at 12 weeks after supplementation versus 76.7% of the placebo group	[47]
TB contacts (192)	London, UK	2.5 mg single dose vitamin D_2_	Those given the vitamin D had enhanced immunity to TB using the lux *in vitro* assays but did not affect IFNg production after ESAT-6/CFP-10 stimulation	[48]
TB patients with pulmonary TB (367, 136 completed trial in vitamin-D-supplemented group, 145 completed in placebo group)	Bissau, Guinea Bissau	100,000 IU of vitamin D given at time of anti-TB treatment, then at 5 and 8 months later	No differences in clinical severity between groups and no differences in mortality 12 months later	[49]
TB (146)	London, UK	2.5 mg vitamin D_2_ given at time of Tb treatment plus 14, 28 and 42 days later	Those on Vitamin D supplementation displayed sputum clearance at 36 days after treatment versus 43.5 dayes but this was not statistically significant but it did significantly hasten sputum culture conversion in participants with the *tt* genotype of the *TaqI* vitamin D receptor polymorphism	[50]

*Paediatric studies*				
Children aged between 1.5 and 13 years of age with TB (24), 13 extra thoracic, 7 intrathoracic, and 4 mixed	Egypt	Vitamin D single dose unable to obtain information regarding* (dosage concentration not reported) *	8 weeks after supplementation greater clinical improvement was observed in vitamin-D-supplemented group	[51]

NB: 25 mg = 1,000 IU.

## References

[1] Nnoaham KE, Clarke A (2008). Low serum vitamin D levels and tuberculosis: a systematic review and meta-analysis. *International Journal of Epidemiology*.

[2] Holick MF (2009). Vitamin D status: measurement, interpretation, and clinical application. *Annals of Epidemiology*.

[3] Vieth R (2011). Vitamin D nutrient to treat TB begs the prevention question. *The Lancet*.

[4] Douglas AS, Strachan DP, Maxwell JD (1996). Seasonality of tuberculosis: the reverse of other respiratory diseases in the UK. *Thorax*.

[5] Holick MF, Binkley NC, Bischoff-Ferrari HA (2011). Evaluation, treatment, and prevention of vitamin D deficiency: an endocrine society clinical practice guideline. *Journal of Clinical Endocrinology and Metabolism*.

[6] Fares A (2011). Seasonality of tuberculosis. *Journal of Global Infectious Diseases*.

[7] Ríos M, García JM, Sánchez JA, Pérez D (2000). A statistical analysis of the seasonality in pulmonary tuberculosis. *European Journal of Epidemiology*.

[8] Schaaf HS, Nel ED, Beyers N, Gie RP, Scott F, Donald PR (1996). A decade of experience with Mycobacterium tuberculosis culture from children: a seasonal influence on incidence of childhood tuberculosis. *Tubercle and Lung Disease*.

[9] Martineau AR, Nhamoyebonde S, Oni T (2011). Reciprocal seasonal variation in vitamin D status and tuberculosis notifications in Cape Town, South Africa. *Proceedings of the National Academy of Sciences of the United States of America*.

[10] Thorpe LE, Laserson K, Cookson S (2004). Infectious Tuberculosis among Newly Arrived Refugees in the United States. *New England Journal of Medicine*.

[11] Strachan DP, Powell KJ, Thaker A, Millard FJC, Maxwell JD (1995). Vegetarian diet as a risk factor for tuberculosis in immigrant south London Asians. *Thorax*.

[12] Hart PD (1946). Chemotherapy of tuberculosis; research during the past 100 years. *British Medical Journal*.

[13] Everett D (1846). On the use of cod-liver oil in tubercular disease. *Provincial Medical Surgical Journal*.

[14] Williams CJB (1849). Cod liver oil in phthisis. *London Journal of Medicine*.

[15] http://www.nobelprize.org/nobel_prizes/medicine/laureates/1903/#.

[16] Hewison M (2010). Vitamin D and the intracrinology of innate immunity. *Molecular and Cellular Endocrinology*.

[17] Rider AA (1970). Elmer Verner McCollum—a biographical sketch (1879–1967). *Journal of Nutrition*.

[18] Charpy J (1945). *Annales de Dermatologie et de Syphiligraphie*.

[19] Gaumond E (1948). Lupus vulgaris and vitamin D. *Canadian Medical Association Journal*.

[20] Dowling GB, Thomas EW, Wallace HJ (1946). *Lupus Vulgaris* treated with Calciferol. *Proceedings of the Royal Society of Medicine*.

[21] Phelan JJ (1947). Calciferol in pulmonary tuberculosis. *The Lancet*.

[22] Martineau AR (2012). Old wine in new bottles: vitamin D in the treatment and prevention of tuberculosis. *Proceedings of the Nutrition Society*.

[23] Zhang R, Naughton DP (2010). Vitamin D in health and disease: current perspectives. *Nutrition Journal*.

[24] Okamura WH, Midland MM, Hammond MW (1995). Chemistry and conformation of vitamin D molecules. *Journal of Steroid Biochemistry and Molecular Biology*.

[25] Wagner CL, Greer FR (2008). Prevention of rickets and vitamin D deficiency in infants, children, and adolescents. *Pediatrics*.

[26] Mimouni FB, Shamir R (2009). Vitamin D requirements in the first year of life. *Current Opinion in Clinical Nutrition and Metabolic Care*.

[27] White JH (2012). Vitamin D metabolism and signaling in the immune system. *Reviews in Endocrine and Metabolic Disorders*.

[28] Singh RJ, Taylor RL, Reddy GS, Grebe SKG (2006). C-3 epimers can account for a significant proportion of total circulating 25-hydroxyvitamin D in infants, complicating accurate measurement and interpretation of vitamin D status. *Journal of Clinical Endocrinology and Metabolism*.

[29] Strathmann FG, Sadilkova K, Laha TJ (2012). 3-epi-25 hydroxyvitamin D concentrations are not correlated with age in a cohort of infants and adults. *Clinica Chimica Acta*.

[30] Fleet JC, Bradley J, Reddy GS, Ray R, Wood RJ (1996). 1*α*,25-(OH)_2_-vitamin D_3_ analogs with minimal in vivo calcemic activity can stimulate significant transepithelial calcium transport and mRNA expression in vitro. *Archives of Biochemistry and Biophysics*.

[31] Molnár F, Sigüeiro R, Sato Y (2011). 1*α*,25(OH)_2_-3-epi-vitamin D_3_, a natural physiological metabolite of vitamin D_3_: its synthesis, biological activity and crystal structure with its receptor. *PLoS ONE*.

[32] Harant H, Spinner D, Reddy GS, Lindley IJD (2000). Natural metabolites of 1*α*,25-dihydroxyvitamin D_3_ retain biologic activity mediated through the vitamin D receptor. *Journal of Cellular Biochemistry*.

[33] Messerlian S, Gao X, St-Arnaud R (2000). The 3-epi- and 24-oxo-derivatives of 1*α*,25 dihydroxyvitamin D_3_ stimulate transcription through the vitamin D receptor. *Journal of Steroid Biochemistry and Molecular Biology*.

[34] Kumar GT, Sachdev HS, Chellani H (2011). Effect of weekly vitamin D supplements on mortality, morbidity, and growth of low birthweight term infants in India up to age 6 months: randomised controlled trial. *British Medical Journal*.

[35] Vieth R (2006). What is the optimal vitamin D status for health?. *Progress in Biophysics and Molecular Biology*.

[36] (1998). Nutrition and bone health: with particular reference to calcium and vitamin D. Report of the Subgroup on Bone Health, Working Group on the Nutritional Status of the Population of the Committee on Medical Aspects of the Food Nutrition Policy. *Reports on Health and Social Subjects*.

[37] Pearce SH, Cheetham TD (2010). Diagnosis and management of vitamin D deficiency. *British Medical Journal*.

[38] Vieth R (2004). Why the optimal requirement for Vitamin D_3_ is probably much higher than what is officially recommended for adults. *Journal of Steroid Biochemistry and Molecular Biology*.

[39] Greer FR (2003). Vitamin D deficiency—it’s more than rickets. *Journal of Pediatrics*.

[40] Ginde AA, Mansbach JM, Camargo CA (2009). Association between Serum 25-hydroxyvitamin D level and upper respiratory tract infection in the Third National Health and Nutrition Examination Survey. *Archives of Internal Medicine*.

[41] Belderbos ME, Houben ML, Wilbrink B (2011). Cord blood vitamin D deficiency is associated with respiratory syncytial virus bronchiolitis. *Pediatrics*.

[42] Fabri M, Stenger S, Shin D-M (2011). Vitamin D is required for IFN-*γ*-mediated antimicrobial activity of human macrophages. *Science Translational Medicine*.

[43] Luxwolda MF, Kuipers RS, Kema IP, Janneke Dijck-Brouwer DA, Muskiet FA Traditionally living populations in East Africa have a mean serum 25-hydroxyvitamin D concentration of 115 nmol/l.

[44] Walker VP, Zhang X, Rastegar I (2011). Cord blood vitamin D status impacts innate immune responses. *Journal of Clinical Endocrinology and Metabolism*.

[45] Hollis BW, Johnson D, Hulsey TC, Ebeling M, Wagner CL (2011). Vitamin D supplementation during pregnancy: double-blind, randomized clinical trial of safety and effectiveness. *Journal of Bone and Mineral Research*.

[46] Introducing Solid Foods- Giving Your Baby a Better Start in Life. http://www.dh.gov.uk/en/Publicationsandstatistics/Publications/PublicationsPolicyAndGuidance/DH_125823.

[47] Nursyam EW, Amin Z, Rumende CM (2006). The effect of vitamin D as supplementary treatment in patients with moderately advanced pulmonary tuberculous lesion. *Acta Medica Indonesiana*.

[48] Martineau AR, Wilkinson RJ, Wilkinson KA (2007). A single dose of vitamin D enhances immunity to mycobacteria. *American Journal of Respiratory and Critical Care Medicine*.

[49] Wejse C, Gomes VF, Rabna P (2009). Vitamin D as supplementary treatment for tuberculosis: a double-blind, randomized, placebo-controlled trial. *American Journal of Respiratory and Critical Care Medicine*.

[50] Martineau AR, Timms PM, Bothamley GH (2011). High-dose vitamin D_3_ during intensive-phase antimicrobial treatment of pulmonary tuberculosis: a double-blind randomised controlled trial. *The Lancet*.

[51] Morcos MM, Gabr AA, Samuel S (1998). Vitamin D administration to tuberculous children and its value. *Bollettino Chimico Farmaceutico*.

[52] Taylor CE, Camargo CA (2011). Impact of micronutrients on respiratory infections. *Nutrition Reviews*.

[53] McNally JD, Leis K, Matheson LA, Karuananyake C, Sankaran K, Rosenberg AM (2009). Vitamin D deficiency in young children with severe acute lower respiratory infection. *Pediatric Pulmonology*.

[54] Laaksi I, Ruohola JP, Tuohimaa P (2007). An association of serum vitamin D concentrations < 40 nmol/L with acute respiratory tract infection in young Finnish men. *American Journal of Clinical Nutrition*.

[55] Roth D, Shah R, Black R, Baqui A (2010). Vitamin D status and acute lower respiratory infection in early childhood in Sylhet, Bangladesh. *Acta Paediatrica, International Journal of Paediatrics*.

[56] Inamo Y, Hasegawa M, Saito K (2011). Serum vitamin D concentrations and associated severity of acute lower respiratory tract infections in Japanese hospitalized children. *Pediatrics International*.

[57] Wayse V, Yousafzai A, Mogale K, Filteau S (2004). Association of subclinical vitamin D deficiency with severe acute lower respiratory infection in Indian children under 5 y. *European Journal of Clinical Nutrition*.

[58] Roth DE, Jones AB, Prosser C, Robinson JL, Vohra S (2009). Vitamin D status is not associated with the risk of hospitalization for acute bronchiolitis in early childhood. *European Journal of Clinical Nutrition*.

[59] Adams JS, Hewison M (2008). Unexpected actions of vitamin D: new perspectives on the regulation of innate and adaptive immunity. *Nature Clinical Practice Endocrinology and Metabolism*.

[60] Lagishetty V, Liu NQ, Hewison M (2011). Vitamin D metabolism and innate immunity. *Molecular and Cellular Endocrinology*.

[61] Provvedini DM, Tsoukas CD, Deftos LJ, Manolagas SC (1983). 1,25-Dihydroxyvitamin D_3_ receptors in human leukocytes. *Science*.

[62] Adorini L, Penna G, Giarratana N (2004). Dendritic cells as key targets for immunomodulation by Vitamin D receptor ligands. *Journal of Steroid Biochemistry and Molecular Biology*.

[63] Kreutz M, Andreesen R, Krause SW, Szabo A, Ritz E, Reichel H (1993). 1,25-Dihydroxyvitamin D_3_ production and vitamin D_3_ receptor expression are developmentally regulated during differentiation of human monocytes into macrophages. *Blood*.

[64] Liu PT, Stenger S, Li H (2006). Toll-like receptor triggering of a vitamin D-mediated human antimicrobial response. *Science*.

[65] Liu PT, Schenk M, Walker VP (2009). Convergence of IL-1*β* and VDR activation pathways in human TLR2/1-induced antimicrobial responses. *PLoS ONE*.

[66] Edfeldt K, Liu PT, Chun R (2010). T-cell cytokines differentially control human monocyte antimicrobial responses by regulating vitamin D metabolism. *Proceedings of the National Academy of Sciences of the United States of America*.

[67] Rook GAW, Steele J, Fraher L (1986). Vitamin D_3_, gamma interferon, and control of proliferation of Mycobacterium tuberculosis by human monocytes. *Immunology*.

[68] Denis M (1991). Killing of Mycobacterium tuberculosis within human monocytes: activation by cytokines and calcitriol. *Clinical and Experimental Immunology*.

[69] Antal AS, Dombrowski Y, Koglin S, Ruzicka T, Schauber J (2011). Impact of vitamin D_3_ on cutaneous immunity and antimicrobial peptide expression. *Dermato-Endocrinology*.

[70] Liu PT, Modlin RL (2008). Human macrophage host defense against Mycobacterium tuberculosis. *Current Opinion in Immunology*.

[71] Yuk JM, Shin DM, Lee HM (2009). Vitamin D_3_ induces autophagy in human monocytes/macrophages via cathelicidin. *Cell Host and Microbe*.

[72] Fabri M, Modlin RL (2009). A vitamin for autophagy. *Cell Host and Microbe*.

[73] Anand SP, Selvaraj P (2009). Effect of 1, 25 dihydroxyvitamin D_3_ on matrix metalloproteinases MMP-7, MMP-9 and the inhibitor TIMP-1 in pulmonary tuberculosis. *Clinical Immunology*.

[74] Jo EK (2010). Innate immunity to mycobacteria: vitamin D and autophagy. *Cellular Microbiology*.

[75] Di Rosa M, Malaguarnera M, Nicoletti F, Malaguarnera L (2011). Vitamin D_3_: a helpful immuno-modulator. *Immunology*.

[76] Yamshchikov AV, Desai NS, Blumberg HM, Ziegler TR, Tangpricha V (2009). Vitamin D for treatment and prevention of infectious diseases: a systematic review of randomized controlled trials. *Endocrine Practice*.

[77] Chen S, Sims GP, Xiao XC, Yue YG, Chen S, Lipsky PE (2007). Modulatory effects of 1,25-dihydroxyvitamin D_3_ on human B cell differentiation. *Journal of Immunology*.

[78] Griffin MD, Xing N, Kumar R (2003). Vitamin D and its analogs as regulators of immune activation and antigen presentation. *Annual Review of Nutrition*.

[79] Mahon BD, Wittke A, Weaver V, Cantorna MT (2003). The targets of vitamin D depend on the differentiation and activation status of CD4 positive T cells. *Journal of Cellular Biochemistry*.

[80] Reichel H, Koeffler HP, Tobler A, Norman AW (1987). 1*α*,25-Dihydroxyvitamin D_3_ inhibits *γ*-interferon synthesis by normal human peripheral blood lymphocytes. *Proceedings of the National Academy of Sciences of the United States of America*.

[81] Cantorna MT, Yu S, Bruce D (2008). The paradoxical effects of vitamin D on type 1 mediated immunity. *Molecular Aspects of Medicine*.

[82] Imazeki I, Matsuzaki J, Tsuji K, Nishimura T (2006). Immunomodulating effect of vitamin D_3_ derivatives on type-1 cellular immunity. *Biomedical Research*.

[83] Vidyarani M, Selvaraj P, Jawahar MS, Narayanan PR (2007). 1, 25 Dihydroxyvitamin D_3_ modulated cytokine response in pulmonary tuberculosis. *Cytokine*.

[84] Daniel C, Sartory NA, Zahn N, Radeke HH, Stein JM (2008). Immune modulatory treatment of trinitrobenzene sulfonic acid colitis with calcitriol is associated with a change of a T helper (Th) 1/Th17 to a Th2 and regulatory T cell profile. *Journal of Pharmacology and Experimental Therapeutics*.

[85] Cantorna MT, Humpal-Winter J, DeLuca HF (2000). *In vivo* upregulation of interleukin-4 is one mechanism underlying the immunoregulatory effects of 1,25-dihydroxyvitamin D_3_. *Archives of Biochemistry and Biophysics*.

[86] Prabhu Anand S, Selvaraj P, Narayanan PR (2009). Effect of 1,25 dihydroxyvitamin D_3_ on intracellular IFN-*γ* and TNF-*α* positive T cell subsets in pulmonary tuberculosis. *Cytokine*.

[87] Boonstra A, Barrat FJ, Crain C, Heath VL, Savelkoul HFJ, O’Garra A (2001). 1*α*,25-Dihydroxyvitamin D_3_ has a direct effect on naive CD4^+^ T cells to enhance the development of Th2 cells. *Journal of Immunology*.

[88] Lange NE, Litonjua A, Hawrylowicz CM, Weiss S (2009). Vitamin D, the immune system and asthma. *Expert Review of Clinical Immunology*.

[89] Black RE, Cousens S, Johnson HL (2010). Global, regional, and national causes of child mortality in 2008: a systematic analysis. *The Lancet*.

[90] Bryce J, Boschi-Pinto C, Shibuya K, Black RE (2005). WHO estimates of the causes of death in children. *The Lancet*.

[91] Walker VP, Modlin RL (2009). The vitamin D connection to pediatric infections and immune function. *Pediatric Research*.

[92] Williams B, Williams AJ, Anderson ST (2008). Vitamin D deficiency and insufficiency in children with tuberculosis. *Pediatric Infectious Disease Journal*.

[93] Altare F, Durandy A, Lammas D (1998). Impairment of mycobacterial immunity in human interleukin-12 receptor deficiency. *Science*.

[94] Watkins MLV, Semple PL, Abel B, Hanekom WA, Kaplan G, Ress SR (2008). Exposure of cord blood to Mycobacterium bovis BCG induces an innate response but not a T-cell cytokine response. *Clinical and Vaccine Immunology*.

[95] Adkins B, Leclerc C, Marshall-Clarke S (2004). Neonatal adaptive immunity comes of age. *Nature Reviews Immunology*.

[96] Levy O (2007). Innate immunity of the newborn: basic mechanisms and clinical correlates. *Nature Reviews Immunology*.

[97] Moyo S, Verver S, Mahomed H (2010). Age-related tuberculosis incidence and severity in children under 5 years of age in Cape Town, South Africa. *International Journal of Tuberculosis and Lung Disease*.

[98] Newton SM, Brent AJ, Anderson S, Whittaker E, Kampmann B (2008). Paediatric tuberculosis. *The Lancet Infectious Diseases*.

[99] Lalor MK, Floyd S, Gorak-Stolinska P (2011). BCG vaccination: a role for vitamin D?. *PLoS ONE*.

[100] Nelson CD, Nonnecke BJ, Reinhardt TA, Waters W, Beitz DC, Lippolis JD (2011). Regulation of mycobacterium-specific mononuclear cell responses by 25-hydroxyvitamin D_3_. *PLoS ONE*.

[101] Gao L, Tao Y, Zhang L, Jin Q (2010). Vitamin D receptor genetic polymorphisms and tuberculosis: updated systematic review and meta-analysis. *International Journal of Tuberculosis and Lung Disease*.

[102] Wilkinson RJ, Llewelyn M, Toossi Z (2000). Influence of vitamin D deficiency and vitamin D receptor polymorphisms on tuberculosis among Gujarati Asians in west London: a case-control study. *The Lancet*.

[103] Bellamy R, Ruwende C, Corrah T (1999). Tuberculosis and chronic hepatitis B virus infection in Africans and variation in the vitamin D receptor gene. *Journal of Infectious Diseases*.

[104] Roth DE, Jones AB, Prosser C, Robinson JL, Vohra S (2008). Vitamin D receptor polymorphisms and the risk of acute lower respiratory tract infection in early childhood. *Journal of Infectious Diseases*.

[105] Manaseki-Holland S, Qader G, Isaq Masher M (2010). Effects of vitamin D supplementation to children diagnosed with pneumonia in Kabul: a randomised controlled trial. *Tropical Medicine and International Health*.

[106] Holick MF (2005). The vitamin D epidemic and its health consequences. *Journal of Nutrition*.

[107] Ziegler TR Impact of Vitamin D Supplementation on Host Immunity to Mycobacterium Tuberculosis and Response to Treatment.

[108] Goswami R, Sharma SK, Mitra DK, Singh UB, Gupta N (2011). *Pulmonary Tuberculosis and Vitamin D*.

[109] Salahuddin N (2011). *Replacement of Vitamin D in Patients With Active Tuberculosis (SUCCINCT)*.

[110] Lolong DB, Ralph AP, Tijtra E, Thio F, Morris P, Maguire G (2011). *L-arginine and Vitamin D Adjunctive therapy in Pulmonary Tuberculosis (TB) (AVDAPT)*.

[111] Davaasambuu G (2010). *Role of Vitamin D in Innate Immunity to Tuberculosis*.

[112] Yim J (2011). *The Impact of Vitamin D on Tuberculosis Among Koreans*.

[113] Mathai D *A Clinical Trial to Study the Effect of the Addition of Vitamin D to Conventional Treatment in New Pulmonary Tuberculosis Patients*.

[114] Moodley A (2011). *Vitamin D Supplementation Enhances Immune Response to Bacille-Calmette-Guerin (BCG) Vaccination in Infants (BCG-25-D)*.

